# Combined anti-tumor necrosis factor-α therapy and DMARD therapy in rheumatoid arthritis patients reduces inflammatory gene expression in whole blood compared to DMARD therapy alone

**DOI:** 10.3389/fimmu.2012.00366

**Published:** 2012-12-04

**Authors:** Carl K. Edwards, Julie S. Green, Hans-Dieter Volk, Michael Schiff, Brian L. Kotzin, Hiroaki Mitsuya, Tatsuya Kawaguchi, Ken-Mei Sakata, John Cheronis, David Trollinger, Danute Bankaitis-Davis, Charles A. Dinarello, David A. Norris, Michael P. Bevilacqua, Mayumi Fujita, Gerd-Rudiger Burmester

**Affiliations:** ^1^Department of Dermatology, University of Colorado Denver, Anschutz Medical CampusAurora, CO, USA; ^2^Department of Rheumatology, University of Colorado Denver, Anschutz Medical CampusAurora, CO, USA; ^3^Campus Charite' Mitte Institute fur Medizinische ImmunologieBerlin, Germany; ^4^Kumamoto University School of MedicineKumamoto, Japan; ^5^Source Precision MedicineBoulder, CO, USA; ^6^Department of Infectious Diseases, University of Colorado Denver, Anschutz Medical CampusAurora, CO, USA; ^7^Denver Veterans Affairs Medical CenterDenver, CO, USA

**Keywords:** gene expression, rheumatoid arthritis, anti-TNF-α, whole blood, biomarker

## Abstract

Periodic assessment of gene expression for diagnosis and monitoring in rheumatoid arthritis (RA) may provide a readily available and useful method to detect subclinical disease progression and follow responses to therapy with disease modifying anti-rheumatic agents (DMARDs) or anti-TNF-α therapy. We used quantitative real-time PCR to compare peripheral blood gene expression profiles in active (“unstable”) RA patients on DMARDs, stable RA patients on DMARDs, and stable RA patients treated with a combination of a disease-modifying anti-rheumatoid drug (DMARD) and an anti-TNF-α agent (infliximab or etanercept) to healthy human controls. The expression of 48 inflammatory genes were compared between healthy controls (*N* = 122), unstable DMARD patients (*N* = 18), stable DMARD patients (*N* = 26), and stable patients on combination therapy (*N* = 20). Expression of 13 genes was very low or undetectable in all study groups. Compared to healthy controls, patients with unstable RA on DMARDs exhibited increased expression of 25 genes, stable DMARD patients exhibited increased expression of 14 genes and decreased expression of five genes, and combined therapy patients exhibited increased expression of six genes and decreased expression of 10 genes. These findings demonstrate that active RA is associated with increased expression of circulating inflammatory markers whereas increases in inflammatory gene expression are diminished in patients with stable disease on either DMARD or anti-TNF-α therapy. Furthermore, combination DMARD and anti-TNF-α therapy is associated with greater reductions in circulating inflammatory gene expression compared to DMARD therapy alone. These results suggest that assessment of peripheral blood gene expression may prove useful to monitor disease progression and response to therapy.

## Introduction

Rheumatoid arthritis (RA) is a chronic, progressive, autoimmune, inflammatory disorder that affects approximately 1% of the population in the United States (Lee and Weinblatt, 2001). Among RA patients, the severity of disease, the spectrum of clinical involvement and the response to therapy vary widely, resulting in significant diagnostic and management challenges. RA is currently monitored via repeated clinical assessment of specific signs and symptoms, and by a variety of blood and radiological tests. Unfortunately, none of the clinical laboratory tests in use today can reliably assess disease activity or predict flares of disease or the extent of underlying joint damage.

RA is characterized by chronic inflammation and hypertrophy of the synovial membranes. Inflammation of the joint occurs in response to production of growth factors, cytokines, and chemokines by many different cell types present in synovium and cartilage, in addition to infiltrating cells from the peripheral blood. Cartilage and bone destruction subsequently occur through the enhanced actions of prostaglandins, leukotrienes, and the matrix degrading metalloproteinases (MMPs). The importance of interleukin-1 (IL-1) and tumor necrosis factor-α (TNF-α) in animal models of RA is well documented (Saklatvala, 1986; Issekutz et al., 1994; Hata et al., 2004). TNF-α is known to stimulate IL-1 and interleukin-6 (IL-6) production in synovial tissue (Pettipher et al., 1986; Brahn et al., 1992). These cytokines enhance migration of inflammatory cells into the joint and stimulate MMP production in synovial fibroblasts and chondrocytes (Pettipher et al., 1986).

Although the most important actions of these proteins are likely to occur in the joint, the joint space is relatively inaccessible, prohibiting quantitative measurement of cytokines. Recent advances in the clinical application of pharmacogenomics suggest that biomarkers of disease activity and drug efficacy can be identified in blood to enable the identification of specific patient populations and to monitor subclinical changes in disease status and responses to treatment over time (Frank and Hargreaves, 2003; McLoughlin et al., 2006; Luo et al., 2011). Changes in the serum levels of several proteins have been observed in RA patients: cytokines such as IL-1α, IL1-β, IL-6, IL-10, and TNF-α are increased in RA compared to normal (Deane et al., 2010; Milman et al., 2010); and intercellular adhesion molecule-1 (ICAM-1) level is increased in juvenile RA compared to normal (Bloom et al., 2005; Ishikawa et al., 2009). Factors such as protein instability and sampling variability may, however, limit analyses of serum protein expression. Because altered gene expression precedes release of cytokines and other immunologically important signaling elements, analysis of specific messenger RNA (mRNA) species associated with these changes will provide the earliest diagnostic signs of disease progression and/or flare. Blood samples from patients with RA have been studied by microarray to profile complex expression patterns of genes contributing to inflammatory joint disease (Bovin et al., 2004; Olsen et al., 2004; Batliwalla et al., 2005; Edwards et al., 2007; van der Pouw Kraan et al., 2007; Ishikawa et al., 2009; Teixeira et al., 2009; Deane et al., 2010).

We have previously shown that among healthy volunteers, expression of a number of inflammatory genes can be accurately measured by quantitative reverse transcriptase PCR (qRT-PCR) from peripheral blood samples collected over time, and that this expression is relatively stable (McLoughlin et al., 2006). Importantly, these data suggest that normal reference ranges can be established for the expression of a broad set of inflammatory genes in human whole blood. Here, we use qRT-PCR to measure inflammatory gene expression in whole blood obtained at a single time point from RA patients with either active (“unstable”) or stable disease on DMARD therapy, and patients with stable disease on combination therapy with a DMARD and anti-TNF-α agent (“combination”), and compared the changes in inflammatory gene expression in these groups to levels in whole blood obtained from healthy controls.

## Materials and methods

### Subject characteristics

Samples from blood donor subjects were collected for the current study with approval of the University of Colorado Institutional Review Board and after obtaining written consent from each volunteer. Whole blood samples were collected at a single time point from 122 apparently healthy blood donors at a local blood bank (Bonfils Blood Center, Denver, CO). Enrollment criteria for blood donors followed the American Red Cross donor standards. Subject age was normally distributed and ranged from 22 to 82 years, with an average age of 47.2 ± 13.1 years. Females (*N* = 63) and males (*N* = 59) were represented in about equal numbers, and 82% of the subjects were Caucasian. RA patients eligible for the study met the following inclusion criteria: (1) diagnosis of RA according to the American College of Rheumatology 1987 diagnostic criteria (Arnett et al., 1988); (2) 18 years of age or older; (3) subjects with “stable” RA as defined by presence or history of moderate to severe RA on stable doses for the previous 3 months of DMARDs, NSAIDs, or oral corticosteroids (≤10 mg/day of prednisolone or equivalent); and (4) subjects with “unstable” active RA as defined by 6 or more swollen joints or 9 or more painful or tender joints at baseline and C-reactive protein (CRP) ≥2 mg/dL at the initial visit and who required more aggressive therapy. Exclusion criteria included: (1) any previous treatment with a non-DMARD immunosuppressive drug; (2) use of any investigational drug or biological agent (except anti-TNF-α therapies) within 3 months prior to enrollment; (3) previous diagnosis of any acute or chronic infectious disease, or with current signs or symptoms of severe, progressive, or uncontrolled systemic disease; (4) pregnancy; (5) history of malignancy in the 5 years prior to study enrollment; or (6) ACR functional class IV. In addition to collection of whole blood samples, physical examination, physician assessment of disease activity and morning stiffness, patient assessment of disease and pain activity, and joint assessment (based on a 66/68-joint count excluding distal interphalangeal joints) were performed at baseline and at 12 weeks after initiation of anti-TNF-α therapy (or continuation of previous DMARD therapy in stable RA patients).

Using the criteria described above, there were three experimental patient subpopulation groups: (1) RA patients who were on a systemic disease-modifying anti-rheumatoid drug (DMARD, either oral prednisone or oral methotrexate), with active disease and judged by their physician to require a change in therapy (“unstable”) (*N* = 18); (2) RA patients receiving DMARD therapy for more than 3 months and judged by their physicians *not* to require a change in therapy (“stable”) (*N* = 26); and (3) clinically stable RA subjects treated for more than 3 months with a DMARD *plus* one of two different anti-TNF-α therapies (infliximab or etanercept) and judged by their physician *not* to require a change in therapy (“combination”) (*N* = 20). Characteristics of each experimental patient group are shown in Table 1.

**Table 1:** **Clinical features of RA patient subpopulation groups tested for qRT-PCR analysis**.

**Clinical feature**	**Unstable on DMARD (*n* = 18)**	**Stable on DMARD (*n* = 26)**	**Stable on combination therapy (*n* = 20)**
Gender (% female)	79	61	85
Age (years)	54.1	55.8	57.7
Race (% white, non-Hispanic)	67	50	90
Duration of disease (months)	220	200	227
DMARD use (%)	75	100	100
Prednisone use (%)	64	75	25
MTX weekly dose (mg)	15.5	9.95	14.5

See “Materials and Methods” section for details on patient background and selection.

### Preparation of nucleic acids and quantitative PCR analysis

Blood was collected from study subjects by standard phlebotomy methods into PAXgene™ tubes (PreAnalytiX, Valencia, CA) to stabilize mRNA levels. Samples were frozen at −70°C and shipped on dry ice in compliance with International Air Transport Association (IATA) shipping regulations. Total RNA was extracted as described previously using the PAXgene™ Blood RNA System (Rainen et al., 2002). The purity and integrity of each RNA sample was determined and the mRNA was converted to cDNA by reverse transcription (Rainen et al., 2002). First-strand cDNA was synthesized from random hexamer-primed RNA templates on the ABI Prism™ 6700 Nucleic Acid Automated Workstation using TaqMan® Reverse Transcription Reagents (Applied Biosystems, Multiscribe #4311235, Foster City, CA), according to the manufacturer's procedure.

Target gene products were analyzed by quantitative PCR of each cDNA preparation using 2X TaqMan® Universal PCR Master Mix (Applied Biosystems, #4305719, Foster City, CA) and Source Precision Medicine's proprietary primer/probe sets and adhering to previously described protocols (McLoughlin et al., 2006). Forty-eight inflammation- and immune-related gene products that were originally selected and verified (McLoughlin et al., 2006) were analyzed with slight modification (Table 2). For example, apoptotic protease activating factor 1 (APAF1) and Cytochrome B-245 beta polypeptide (CYBB) were replaced with transforming growth factor beta 1 (TGFB1, a pro- and anti-inflammatory cytokine) and vascular endothelial growth factor (VEGF, an inducer of angiogenesis) because of their involvement in inflammation and the pathogenesis of RA (Kasama et al., 2001; Mattey et al., 2005). Reactions were run in four replicates on an ABI Prism 7700 Sequence Detection System. The amount of cDNA added to each reaction was held to a narrow range, within 1.5 C_T_'s, based on the threshold cycle (C_T_) of the 18S RNA control reaction. The coefficient of variation in the C_T_ values detected for each gene loci were less than 2% in a study that consisted of more than 1000 repeat analyses of the same freezer-stored sample. The repeat analyses were performed over greater than a 2-year period.

**Table 2:** **List of 48 inflammatory genes analyzed in the study and mean ΔCT of gene expression in healthy subjects**.

**HUGO designation**	**Gene name**	**Gene function**	**Mean ΔCT of healthy subjects (*n* = 122)**
B7	CD80	Regulatory protein that may be associated with lupus	21.17
C1QA	Complement component 1, q subcomponent, A chain	Serum complement system component, forms C1 complex with pro-enzymes C1r and C1s	21.64
CD3Z	CD247 molecule	T-cell surface glycoprotein	15.20
CD4	CD4 molecule	Helper T-cell marker; accessory protein in the MHC/T-cell receptor interaction	15.84
CD8A	CD8a molecule	Cytotoxic/suppressor T-cell marker; binds MHC I; thought to play role T-cell mediated killing	16.40
CD14	CD14 molecule	Monocyte LPS receptor; cooperates with MD-2 and TLR-4 in response to LPS	14.84
CD19	CD19 molecule	B-cell growth factor; membrane protein that potentiates receptor-dependent activation	18.65
CXCL1	Chemokine (C-X-C motif) ligand 1	Chemotactic for neutrophils; pro-inflammatory; modulates metalloproteinase activity	19.78
CXCL2	Chemokine (C-X-C motif) ligand 2	Chemotactic for neutrophils and hematopoietic precursor cells	23.32
CSF2	Colony stimulating factor 2	*aka* GM-CSF; stimulates growth and differentiation of hematopoietic precursor cells	23.59
CSF3	Colony stimulating factor 3	*aka* GCSF; cytokine that stimulates granulocyte development	23.45
F3	Coagulation factor 3 (thromboplastin, tissue factor)	*aka* thromboplastin, coagulation factor 3; responsible for coagulation catalysis	23.63
HLA-DRB1	Major histocompatibility complex, class II, DR beta 1	Binds antigen for presentation to CD4^+^ cells	22.09
HMOX1	Heme oxygenase (decycling) 1	Essential for heme catabolism; cleaves heme to form biliverdin and CO; endotoxin inducible	17.10
HSPA1A	Heat shock protein A1A	Molecular chaperone; stabilizes AU rich mRNA; hydrophobic peptide	14.76
ICAM1	Intercellular adhesion molecule 1	Endothelial cell surface molecule; regulates cell adhesion and trafficking of leukocytes	18.05
IFNA2	Interferon, alpha 2	Interferon produced by macrophages with antiviral effects	22.20
IFNG	Interferon gamma	Pro- and anti-inflammatory activity, TH_1_ cytokine, inflammatory mediator of activated T-cells	22.98
IL1A	Interleukin-1, alpha	Pro-inflammatory; generally cytosolic, released during severe inflammatory disease	23.14
IL1B	Interleukin-1, beta	Pro-inflammatory; made by activated macrophages; endogenous pyrogen	16.19
IL1RN	Interleukin-1 receptor antagonist	Anti-inflammatory; inhibits binding of IL-1 its receptor without stimulating IL-1 activity	16.41
IL2	Interleukin-2	T-cell growth factor, expressed by activated T-cells, TH_1_ cytokine	23.11
IL4	Interleukin-4	Anti-inflammatory; TH_2_; suppresses cytokines, increases IL-1RN expression	23.25
IL5	Interleukin-5	Stimulates eosinophil expansion and B-cell differentiation	22.70
IL6	Interleukin-6	Pro- and anti-inflammatory activity; TH_2_ cytokine; regulates hematopoiesis	23.11
IL8	Interleukin-8	Pro-inflammatory CXC chemokine; major secondary inflammatory mediator	21.04
IL10	Interleukin-10	Anti-inflammatory; TH_2_ cytokine; suppresses production of pro-inflammatory cytokines	22.84
IL12B	Interleukin-12b	Pro-inflammatory, TH_1_ cytokine, requires co-stimulation with IL-18 to induce IFN-γ	23.28
IL13	Interleukin-13	Inhibits inflammatory cytokine production	23.09
IL15	Interleukin-15	Pro-inflammatory; T-cell activator; inhibits apoptosis; with IL-2 induces IFN-γ and TNF-α	22.05
IL18	Interleukin-18	Pro-inflammatory; TH_1_ cytokine; promotes apoptosis; induces IFNγ ; blocked by IL18BP	20.12
IL18BP	Interleukin-18 binding protein	Binds and inactivates IL18; implicated in inhibition of early TH_1_ cytokine responses	17.59
MMP3	Matrix metallopeptidase 3	*aka* stromelysin; degrades fibronectin, laminin, and gelatin	23.71
MMP9	Matrix metallopeptidase 9	Degrades extracellular matrix molecules; made by neutrophils; Role in arthritis and metastasis	16.39
PLA2G7	Phospholipase A2, group VII	Activates platelet activating factor (PF4)	19.93
NOS2A	Nitric oxide synthase 2a, inducible	*aka* iNOS; produces NO which is bacteriocidal/tumoricidal	23.53
PLAUR	Plasminogen activator, urokinase receptor	Ligand-specific cell surface receptor for UPA; localizes and promotes plasmin formation	15.59
PTGS2	Prostaglandin-endoperoxide synthase 2	Pro-inflammatory; regulates angiogenesis and cell migration	17.92
PTPRC	Protein tyrosine phosphatase, receptor type, C	An essential regulator of T- and B-cell antigen receptor signaling; suppresses JAK kinases	11.89
SERPINE1	Serpin peptidase inhibitor, clade E	Interacts with tissue plasminogen activator to regulate fibrinolysis; inhibits PLAU	22.95
TGFB1	Transforming growth factor, beta 1	Pro- and anti-inflammatory activity; anti-apoptotic	13.55
TIMP1	TIMP metallopeptidase inhibitor 1	Inhibitors of matrix metalloproteinases; transcriptionally induced by cytokines and hormones	15.11
TNF	Tumor necrosis factor	Pro-inflammatory TH_1_ cytokine, primary mediator of immune response and regulation	20.65
TNFSF5	CD40 ligand	Ligand for CD40; expressed on T-cells; regulates B-cell function by engaging CD40	17.61
TNFSF6	Fas ligand (TNF superfamily, member 6)	Ligand for FAS antigen; critical in triggering apoptosis	21.00
TNFSF13B	Tumor necrosis factor (ligand) superfamily, member 13b	B cell activating factor, TNF family	15.46
TNFRSF13B	Tumor necrosis factor receptor superfamily, member 13b	Controls T-cell-dependent B-cell antibody responses	20.81
VEGF	Vascular endothelial growth factor	Produced by monocytes	23.07

### Data analysis

Each PCR reaction contained primer/probe sets for the target gene and 18S RNA, used as the internal control. The difference between the fluorescence C_T_ for the target and the internal endogenous control (18S) is presented as a ΔC_T_ value. Increases or decreases in the target mRNA concentration correspond to lower or higher ΔC_T_ values, respectively, at approximately 2-fold concentration change per ΔC_T_ unit. The C_T_ reporting system and estimation of relative gene expression is well described in the literature (Livak and Schmittgen, 2001). ΔC_T_ values above 23 should be interpreted with caution, because they correspond to gene expression levels at or below the linear range of the assay. Statistical measures were determined using Enterprise Guide version 2.05.89 (SAS Institute, Inc., Cary, NC). The Anderson-Darling test and the Shapiro-Wilk test were used to determine whether the gene expression data fit a normal distribution. Student's *t*-tests were performed to determine *P* values.

## Results

### Altered gene expression in unstable RA patients compared to healthy subjects

**Figure 1:**
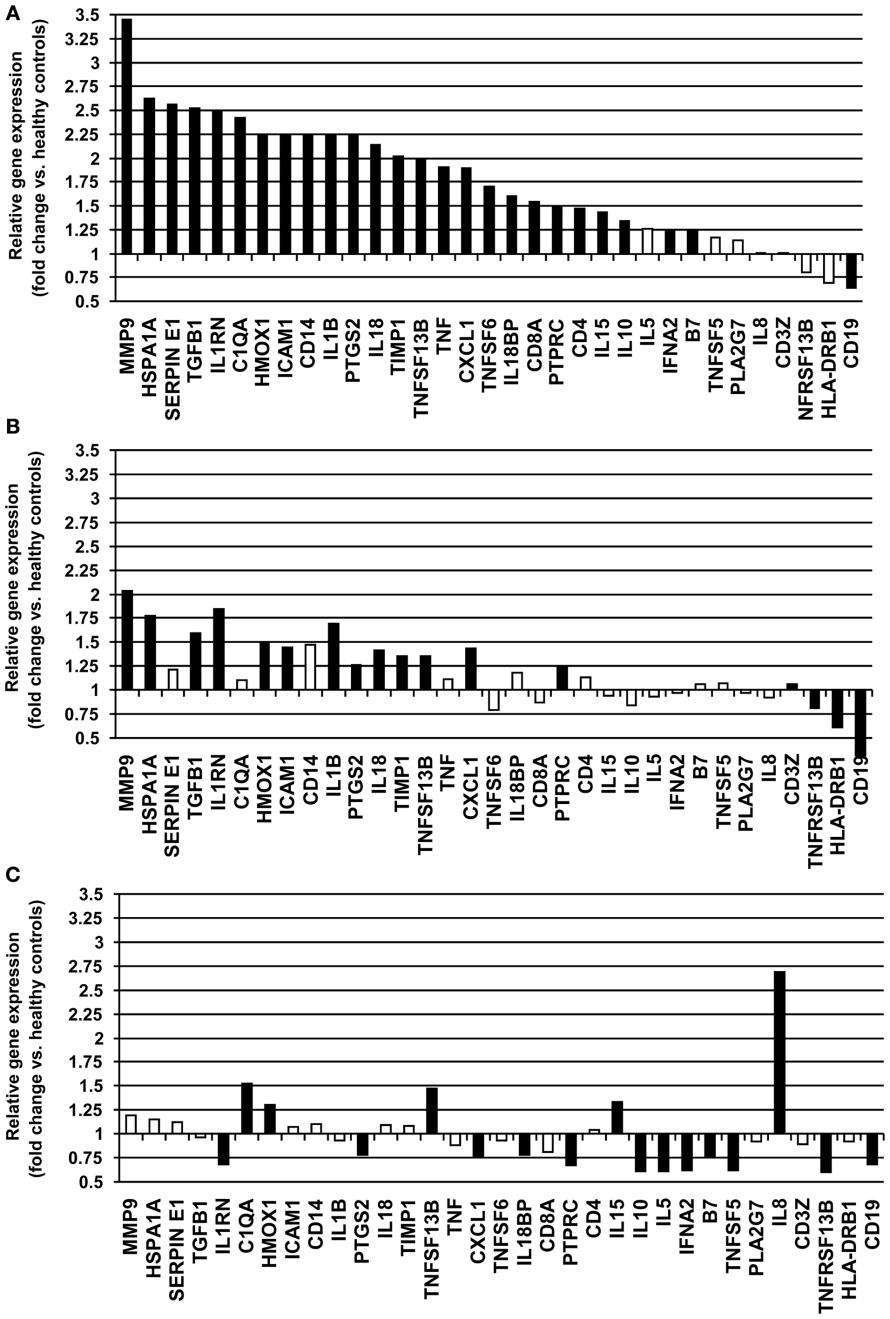
**Relative fold-changes in whole blood gene expression in RA patients compared to healthy controls. (A)** Unstable RA patients on DMARD therapy (*n* = 18). **(B)** Stable RA patients on DMARD therapy (*n* = 26). **(C)** Stable RA patients on combined DMARD and anti-TNF-α therapy (*n* = 20). Changes in expression for each individual gene are averaged and compared to healthy controls. Significant changes in gene expression (*P* < 0.05) are depicted as closed bars, and non-significant changes in gene expression are depicted as open bars.

Gene expression levels were measured in whole blood samples collected from 18 unstable patients with RA maintained on DMARD therapy. Of a total of 48 gene products analyzed, 13 demonstrated very low or undetectable levels among the study subjects. These genes (CSF2, CSF3, CXCL2, F3, IL1A, IL2, IL4, IL6, IL12B, IL13, MMP3, NOS2A, and PLAUR) were not included in further analysis. Of 35 inflammation-related genes examined, these unstable RA patients exhibited increased expression of 25 genes (B7, C1QA, CD14, CD19, CD4, CD8A, CXCL1, HMOX1, HSPA1A, ICAM1, IL10, IL15, IL18, IL18BP, IL1RN, IL1B, MMP9, PTGS2, PTPRC, SERPINE1, TGFB1, TIMP1, TNF, TNFSF13B, TNFSF6, and VEGF) and decreased expression of 1 gene (CD19) compared to healthy controls (*P* < 0.05) (Table 3, Figure 1A). MMP9, HSPA1A, SERPINE1, and TGFB exhibited the greatest increases in mean level of expression in RA patients compared to healthy subjects. Changes in expression of each individual gene averaged over all patients in the unstable RA patient group and compared to average gene expression in the healthy control population are depicted in Figure 1A.

**Table 3:** **Single time point gene expression analysis in RA patients unstable on DMARD therapy, stable on DMARD therapy, and stable on combination therapy**.

**Gene name**	**Relative expression (fold change)^a^**			***P* value^b^**		
	**Unstable on DMARD (*n* = 18**)	**Stable on DMARD (*n* = 26**)	**Stable on anti-TNF (*n* = 20**)	**Unstable on DMARD (*n* = 18**)	**Stable on DMARD (*n* = 26**)	**Stable on anti-TNF (*n* = 20**)
B7	1.24	1.07	0.75	**0.0475**	0.5217	**0.0100**
C1QA	2.42	1.10	1.52	<**0.0001**	0.5567	**0.0251**
CD14	2.25	1.47	1.10	<**0.0001**	**0.0006**	0.3615
CD19	0.64	0.54	0.68	**0.0090**	<**0.0001**	**0.0132**
CD3Z	1.01	0.81	0.89	0.9495	**0.0160**	0.1683
CD4	1.47	1.13	1.04	**0.0012**	0.2490	<**0.0001**
CD8A	1.54	0.87	0.81	**0.0062**	0.3299	0.1707
CXCL1	1.89	1.43	0.75	<**0.0001**	**0.0016**	**0.0263**
HLA-DRB1	0.69	0.32	0.92	0.4900	**0.0379**	0.9067
HMOX1	2.25	1.49	1.30	<**0.0001**	**0.0003**	**0.0385**
HSPA1A	2.62	1.77	1.15	<**0.0001**	<**0.0001**	0.2548
ICAM1	2.25	1.44	1.07	<**0.0001**	**0.0003**	0.5007
IFNA2	1.25	1.06	0.62	0.1295	0.7230	**0.0004**
IFNG	1.24	0.84	ND	0.0614	0.0795	^*^
IL10	1.34	0.93	0.61	**0.0206**	0.5005	<**0.0001**
IL15	1.43	0.94	1.33	**0.0095**	0.6242	**0.0447**
IL18	2.14	1.41	1.09	<**0.0001**	**0.0010**	0.4815
IL18BP	1.60	1.18	0.78	<**0.0001**	0.0644	**0.0025**
IL1RN	2.48	1.84	0.68	<**0.0001**	<**0.0001**	**0.0006**
IL1B	2.24	1.69	0.93	<**0.0001**	<**0.0001**	0.5218
IL5	1.26	0.97	0.61	0.0857	0.8539	<**0.0001**
IL8	1.01	1.06	2.69	0.9721	0.7469	<**0.0001**
MMP9	3.45	2.04	1.19	<**0.0001**	**0.0001**	0.3469
PLA2G7	1.14	0.92	0.92	0.4591	0.5298	0.6262
PTGS2	2.23	1.26	0.78	<**0.0001**	**0.0248**	**0.0136**
PTPRC	1.49	1.25	0.67	<**0.0001**	**0.0138**	0.6788
SERPINE1	2.56	1.21	1.12	<**0.0001**	0.1680	0.4726
TGFB1	2.52	1.59	0.96	<**0.0001**	<**0.0001**	0.5757
TIMP1	2.02	1.35	1.08	<**0.0001**	**0.0010**	0.4304
TNF	1.90	1.11	0.88	<**0.0001**	0.3426	0.3158
TNFRSF13B	0.80	0.61	0.60	0.1674	**0.0004**	**0.0028**
TNFSF13B	1.97	1.35	1.47	<**0.0001**	**0.0137**	**0.0017**
TNFSF5	1.17	0.97	0.62	0.1691	0.7831	<**0.0001**
TNFSF6	1.70	0.79	0.93	**0.0008**	0.0839	0.6686
VEGF	1.38	0.84	ND	**0.0028**	**0.0474**	^*^

a ΔCT values of genes expressed in RA subjects were compared to those from healthy blood donors.

b Probability of a difference between groups was determined by Student's t-test. P value represents comparison of gene expression from healthy blood donors.

ND indicates data not determined.

* Indicates statistics not analyzed.

Bold values are statistically significant P values.

### Changes in gene expression during DMARD therapy

Whole blood was collected from 26 RA patients with stable clinical examinations after at least 12 weeks of DMARD therapy. Serum levels of CRP in these patients ranged from 0.05 to 2.7 mg/L (average 0.35 mg/L), consistent with low systemic levels of inflammation. Of 35 inflammation-related genes examined, stable RA patients maintained on DMARD therapy exhibited increased expression of 14 genes (CD14, CXCL1, HMOX1, HSPA1A, ICAM1, IL18, IL1RN, IL1B, MMP9, PTGS2, PTPRC, TGFB1, TIMP1, and TNFSF13B) and decreased expression of 5 genes (CD19, CD3Z, HLA-DRB1, TNFRSF13B, and VEGF) compared to healthy controls (*P* < 0.05) (Table 3, Figure 1B). Expression of B7, C1QA, CD4, CD8A, IL10, IL15, IL18BP, SERPINE1, TNF, and TNFSF6 were normalized compared to unstable RA patients maintained on DMARD therapy (Figures 1A,B).

### Changes in gene expression during combination therapy

Whole blood was collected from 20 RA patients with stable clinical examinations after 12 weeks of treatment with combination therapy (a DMARD and an anti-TNF-α agent). Of 35 inflammation-related genes examined, 6 genes (C1QA, CD4, HMOX1, IL15, IL8, and TNFSF13B) exhibited increased expression and 11 genes (B7, CD19, CXCL1, IFNA2, IL10, IL18BP, IL1RN, IL5, PTGS2, TNFRSF13B, and TNFSF5) demonstrated decreased expression compared to healthy controls (*P* < 0.05) (Table 3, Figure 1C). The increased expression of 15 genes present in the unstable RA patient group on DMARD therapy was normalized in stable RA patients on combination therapy (Figures 1A,C). These genes included CD14, CD8A, HSPA1A, ICAM1, IL18, IL1B, MMP9, PTPRC, SERPINE1, TGFB1, TIMP1, TNF, and TNFSF6.

Among 20 patients receiving combination therapy, patients receiving infliximab (*N* = 10) exhibited decreased expression of seven genes (B7, CXCL1, IL10, IL1RN, IL5, PTPRC, and TNFSF5) and increased gene expression of one gene (C1QA) compared to healthy controls, whereas patients receiving etanercept (*N* = 10) exhibited increased expression of three genes (HMOX1, IL8, and TNFSF13B) and decreased expression of nine genes (CD8A, IL10, IL18BP, IL1RN, IL5, PTGS2, PTPRC, TNFRSF13B, and TNFSF5 compared to healthy controls (*P* < 0.05) (Table 4). RA patients stable on etanercept exhibited robust increases in IL8 expression (5.70-fold change compared to healthy controls, *P* < 0.0001, Table 4).

**Table 4:** **Single time point gene expression analysis of stable RA patients on DMARD therapy and anti-TNF-α therapy with either infliximab or etanercept**.

**Gene name**	**Relative expression (fold change)^a^**		***P* value^b^**	
	**Stable on infliximab (*n* = 10**)	**Stable on etanercept (*n* = 10**)	**Stable on infliximab (*n* = 10**)	**Stable on etanercept (*n* = 10**)
B7	0.67	0.92	**0.0024**	0.4999
C1QA	2.02	0.99	**0.0014**	0.9696
CD14	1.14	1.04	0.3689	0.7978
CD3Z	0.89	0.94	0.2948	0.5865
CD4	1.03	1.06	0.8423	0.6788
CD8A	1.04	0.58	0.8418	**0.0063**
CXCL1	0.68	0.79	**0.0284**	0.1727
HMOX1	1.26	1.40	0.1278	**0.0451**
HSPA1A	1.15	1.09	0.3865	0.5708
ICAM1	0.98	1.13	0.8911	0.3179
IL10	0.68	0.64	**0.0183**	**0.003**
IL15	1.43	1.20	0.0555	0.3005
IL18	0.90	1.25	0.4656	0.0925
IL18BP	0.82	0.73	0.0526	**0.003**
IL1RN	0.61	0.73	**0.0014**	**0.0203**
IL1B	0.78	1.07	0.078	0.6039
IL5	0.66	0.46	**0.0101**	<**0.0001**
IL8	1.50	5.70	0.1585	<**0.0001**
MMP9	1.20	1.06	0.461	0.7911
PLA2G7	0.86	1.07	0.4403	0.7478
PTGS2	0.82	0.71	0.1617	**0.012**
PTPRC	0.75	0.63	**0.0123**	<**0.0001**
SERPINE1	1.21	0.94	0.286	0.7372
TGFB1	1.02	0.88	0.8391	0.1836
TIMP1	1.02	1.10	0.8459	0.4071
TNF	0.83	0.81	0.2149	0.1582
TNFRSF13B	0.76	0.51	0.1389	**0.0004**
TNFSF13B	1.37	1.49	0.0592	**0.0117**
TNFSF5	0.59	0.62	**0.0002**	**0.001**
TNFSF6	1.09	0.75	0.6164	0.102

a ΔCT values of genes expressed in RA subjects were compared to those from healthy blood donors.

b Probability of a difference between groups was determined by Student's t-test. P value represents comparison of gene expression from healthy blood donors.

Bold values are statistically significant P values.

## Discussion

Identification of easily accessible and reliable biomarkers of inflammatory disease activity to diagnose and monitor disease progression in individual patients over time is an attractive therapeutic goal. Here, we demonstrate that gene expression analysis of whole blood using qRT-PCR can be used to assess disease activity of RA patients.

Patients with unstable RA demonstrated increased peripheral blood expression of numerous proinflammatory cytokines, including IL1B, TNF, and IL18, and increased expression of genes whose protein products have been shown to contribute to synovial deterioration (including MMP) compared to healthy human control subjects. These cytokines have been previously shown to be upregulated in the synovium or serum in RA (Brennan et al., 1992; Gracie et al., 1999; Feldmann and Maini, 2001; Joosten et al., 2004; Klimiuk et al., 2004; Paramalingam et al., 2007; Shao et al., 2009; Volin and Koch, 2011). In comparison, increases in inflammatory gene expression are diminished in patients with stable disease on either DMARD alone or combined DMARD and anti-TNF-α therapy. These results indicate that clinical assessments of overall disease stability correlate with an overall reduction in peripheral inflammatory gene expression.

Unstable RA patients in the current study also exhibited increased expression of several cytokines which are generally thought to have an anti-inflammatory effect, including IL10, IL1RN, and TGFB1 (Katsikis et al., 1994). These have been reported to be elevated in the synovium and in peripheral blood samples from RA patients (Katsikis et al., 1994; Ohshima et al., 1999; Mesko et al., 2010; Tukaj et al., 2010; Meugnier et al., 2011), where they may serve to mitigate the inflammatory process. Further, expression of IL10 and IL1RN in stable RA patients on either DMARD or combination DMARD and anti-TNF-α therapy was the same (DMARD) or reduced (combination) compared to healthy controls (Table 3). It is speculated that as levels of proinflammatory cytokines are reduced during effective anti-TNF-α therapy, corresponding levels of anti-inflammatory cytokines are also reduced.

Our data, however, also demonstrate that despite stable clinical assessments, RA patients on DMARD therapy alone continue to exhibit increased expression of some inflammatory genes, suggesting that subclinical inflammation is still present in these patients. Stable RA patients on combination therapy demonstrate a greater reduction in inflammatory gene expression compared to both unstable and stable RA patients on DMARD alone. Specifically, patients on combination therapy exhibit fewer increases in proinflammatory markers compared to patients on DMARD alone, and decreased expression of a number of proinflammatory genes even compared to healthy controls. Our findings suggest that even when chronic inflammatory disease is clinically stable, inflammatory pathways may still be active. These pathways could contribute to long-term development of comorbidities associated with RA. Further, variations in the inflammatory profile differ in patients on DMARD alone versus combined DMARD and anti-TNF-α therapy, and combination therapy appears to confer a greater overall anti-inflammatory effect compared to DMARD therapy. Whether changes in the expression of particular genes may be useful biomarkers to assess overall disease activity remains to be determined, and is an area of ongoing research.

Changes in gene expression with anti-TNF-α therapy were largely similar between patients receiving infliximab and etanercept, with the notable exception of significantly increased expression of the chemokine IL8 in patients on etanercept compared to healthy controls and to RA patients on DMARD or infliximab therapies (Table 3). IL8 recruits neutrophils and T cells to the synovium and stimulates angiogenesis (reviewed in Badolato and Oppenheim, 1996). The mechanisms and long-term consequences of IL8 induction during anti-TNF-α treatment are unclear and further studies are warranted.

Despite variable disease activity and treatment modalities, the expression of several proinflammatory genes was increased among all three experimental groups compared to healthy controls, including HMOX1 and TNFSF13B (Table 3). HMOX1 is a heme oxygenase that reduces levels of the pro-oxidant heme to reduce overall oxidative stress. Its expression is induced by a variety of noxious stimuli, including hypoxia, inflammation, and environmental stressors (Otterbein et al., 2003). TNFSF13B is increased by TNF-α and IFN-γ in the synovium (Alsaleh et al., 2007; Assi et al., 2007; Woo et al., 2011). Sustained elevations of these genes may be associated with the inflammation in the synovium and serum during the development and progression of RA. TNFSF13B is also known as B-cell activating factor (BAFF). Increased TNFSF13B can be contrasted with the expression of CD19, a marker of B cells, which was consistently suppressed relative to healthy controls among all three RA groups, as has been previously reported (Holden et al., 2011).

Assessment of peripheral blood gene expression provides an easily accessible population of inflammatory cells in which to study relative changes in cytokine expression over time. However, it is important to consider that in any given individual, relative proportions of each blood cell type may vary markedly. In this setting, overall changes in peripheral blood gene expression may be significantly influenced by changes in the proportion of blood cell types and their corresponding transcription profiles. For example, we observed upregulation of CD14, a monocyte-specific marker, in unstable RA patients treated with DMARD (Table 3). CD14 expression was attenuated in stable RA patients on DMARD therapy and was normalized in stable RA patients on anti-TNF-α therapy (Table 3). In our unstable RA patient population, therefore, an increased proportion of circulating monocytes may have influenced the expression levels of some cytokines.

IL-6 is a proinflammatory cytokine that stimulates differentiation of B-cells into antibody-producing plasma cells and contributes to the release of metalloproteases from tissue fibroblasts (Badolato and Oppenheim, 1996). Increases in IL-6 protein levels in the synovium and the serum have been demonstrated in RA patients, particularly associated with acute inflammation early in the disease process (Houssiau et al., 1988; Hovdenes et al., 1990; Madhok et al., 1993). Our results demonstrate very low or undetectable levels of IL6 mRNA from peripheral blood cells of stable RA patients treated with DMARD therapy and in stable RA patients treated with combination DMARD and anti-TNF-α therapy, suggesting significant attenuation of the proinflammatory cytokine cascade that contributes to the progression of RA. Expression of IL-6 is tightly regulated, and changes in gene transcription may precede measurable changes in protein secretion (DeFuego and Remick, 1991). High serum levels of IL-6 despite low levels of IL6 mRNA expression within mononuclear cells isolated from the peripheral blood and from the synovial fluid in RA patients suggest that high circulating proinflammatory cytokine levels do not necessarily reflect increases in gene expression within inflammatory cell populations (Vazquez-Del Mercado et al., 1999). Our study examines mRNA levels of IL6, which may not correspond to circulating levels of the protein product. IL-6 within the joint space is derived from synovial fibroblasts (Park and Pillinger, 2007; Verweij and Vosslamber, 2012); high levels of circulating IL-6 may derive from tissues rather than from circulating mononuclear cells. Finally, previous studies have shown no differences in IL-6 protein or mRNA transcript levels between early, untreated RA patients and chronic RA patients previously treated with DMARD (Vazquez-Del Mercado et al., 1999). Methotrexate, for example, has been shown to inhibit IL-6 production in peripheral blood cells (Aggarwal and Misra, 2003). All three patient groups in the current study were treated with DMARD therapy which may suppress gene expression of IL6 even in the setting of active clinical disease. Finally, circulating levels of IL-6 follow a circadian rhythm in both healthy subjects and RA patients (Knudsen et al., 2008; Perry et al., 2009); differences in sampling time between patients in this study may have impacted our measurement of IL6 levels in the overall study population. Further investigation regarding the significance of low levels of IL6 in these unstable patients is warranted.

Herein, we show that reduction of inflammatory gene expression levels differ between stable patients on DMARD and combined DMARD and anti-TNF-α therapies. Since most genes in healthy subjects exhibit limited dynamic ranges of expression, we believe that in our study populations, differences in gene expression levels may reflect disease activity. The current findings suggest that these peripheral blood biomarkers correlate with clinical status, and may therefore provide adjunctive information about the efficacy of various treatments for RA. These biomarkers may also allow for assessment of the efficacy of particular RA treatments, including anti-TNF-α therapies. Future studies to determine peripheral blood biomarkers that may predict individual patient responses to a particular systemic therapy may provide assistance with clinical decision making.

### Conflict of interest statement

The authors have read the journal's policy and have the following conflicts. John Cheronis, David Trollinger, Danute Bankaitis-Davis, and Michael Bevilacqua are employees of Source MDx. Source MDx only helped to carry out high-throughput qRT-PCR analysis of whole blood samples. The company was not involved in sample collection and data interpretation, decision to publish, or preparation of the manuscript. This does not alter the authors' adherence to all the *Frontiers in Inflammation* policies on sharing data and materials.
